# Oxygen-Independent Stabilization of Hypoxia Inducible Factor (HIF)-1 during RSV Infection

**DOI:** 10.1371/journal.pone.0003352

**Published:** 2008-10-07

**Authors:** Helene A. Haeberle, Carin Dürrstein, Peter Rosenberger, Yashoda M. Hosakote, Johannes Kuhlicke, Volkhard A. J. Kempf, Roberto P. Garofalo, Holger K. Eltzschig

**Affiliations:** 1 Department of Anesthesiology and Intensive Care Medicine, University of Tübingen, Tübingen, Germany; 2 Medical Microbiology and Hygiene Department, University of Tübingen, Tübingen, Germany; 3 Department of Pediatrics, University of Texas Medical Branch, Galveston, Texas, United States of America; 4 Mucosal Inflammation Program, Department of Anesthesiology and Perioperative Medicine, University of Colorado Health Science Center, Denver, Colorado, United States of America; University of Giessen Lung Center, Germany

## Abstract

**Background:**

Hypoxia-inducible factor 1 (HIF)-1α is a transcription factor that functions as master regulator of mammalian oxygen homeostasis. In addition, recent studies identified a role for HIF-1α as transcriptional regulator during inflammation or infection. Based on studies showing that respiratory syncytial virus (RSV) is among the most potent biological stimuli to induce an inflammatory milieu, we hypothesized a role of HIF-1α as transcriptional regulator during infections with RSV.

**Methodology, Principal Findings:**

We gained first insight from immunohistocemical studies of RSV-infected human pulmonary epithelia that were stained for HIF-1α. These studies revealed that RSV-positive cells also stained for HIF-1α, suggesting concomitant HIF-activation during RSV infection. Similarly, Western blot analysis confirmed an approximately 8-fold increase in HIF-1α protein 24 h after RSV infection. In contrast, HIF-1α activation was abolished utilizing UV-treated RSV. Moreover, HIF-α-regulated genes (VEGF, CD73, FN-1, COX-2) were induced with RSV infection of wild-type cells. In contrast, HIF-1α dependent gene induction was abolished in pulmonary epithelia following siRNA mediated repression of HIF-1α. Measurements of the partial pressure of oxygen in the supernatants of RSV infected epithelia or controls revealed no differences in oxygen content, suggesting that HIF-1α activation is not caused by RSV associated hypoxia. Finally, studies of RSV pneumonitis in mice confirmed HIF-α-activation in a murine in vivo model.

**Conclusions/Significance:**

Taking together, these studies suggest hypoxia-independent activation of HIF-1α during infection with RSV in vitro and in vivo.

## Introduction

A number of elegant studies, exemplified by those defining induction of the erythropoietin (EPO) gene [1], [2], have utilized multidisciplinary approaches to elucidate basic hypoxia-adaptive responses. Today, convincing evidence confirms a central role of hypoxia-inducible factor (HIF)-1 in mammalian oxygen homeostasis [3]–[6]. Such studies demonstrated that HIF-1 is composed of two subunits: constitutively expressed HIF-1β and oxygen-regulated HIF-1α. Under normoxic conditions, HIF-1α is subjected to hydroxylation on proline residues [7]. The modification is required for the binding of the von Hippel-Lindau (VHL) tumor suppressor protein, the recognition component of an E3 ubiquitin-protein ligase that targets HIF-1α for proteasomal degradation. Under hypoxic conditions, hydroxylation is inhibited and the VHL protein does not bind to HIF-1, eventually leading to stabilization of the alpha-subunit, heterodymerization, nuclear translocation and transcription of HIF-dependent genes.

For example, binding of HIF-1 to consensus domains in the erythropoietin promoter results in the transcriptional induction of HIF-1-bearing gene promoters [8]. A series of experiments by Wang and Semenza [9]–[11] and Maxwell *et al.* [12] demonstrated that reporter genes containing the erythropoetin enhancer were induced by hypoxia in a variety of cell types that did not normally produce erythropoitin. Subsequently, it was determined that HIF-1 is widely expressed and that consensus HIF-1 binding sequences exist in a number of genes other than that of erythropoitin, and were termed hypoxia responsive elements (HRE [8]). In particular, HIF-1 has been found to regulate multiple genes that include HRE in their promoter region, including vascular endothelial growth factor (VEGF), insulin-like growth factors (IGFs), their binding proteins [insuline-like growth factor binding proteins (IGFBPs)] and iron supply regulating genes [e.g. transferrin [13]]. Thus, the discovery of HIF-1 represented a major advance in the understanding of gene regulation by hypoxia. Such studies have led to an understanding that induction of HIF-1 responsive genes drives altered cellular metabolism, increased vascular mass and diameter and increased oxygen carrying capacity of the blood; all events which are conducive to an adaptive response to diminished oxygen supply [1], [14]–[16].

However, studies that are more recent have identified an additional role of HIF-1α as transcriptional regulator of inflammation and infection. For example, HIF-1α is essential for myeloid cell-mediated inflammation, bactericidal capacity of phagocytes [17] and mice with conditional knockouts of HIF-1α show profound impairment of myeloid cell aggregation, motility, invasiveness, and bacterial killing [18]. Moreover, studies of HIF-1α during infection with enterobacteriaceae revealed hypoxia-independent activation by bacterial siderophores [19]. Similarly, HIF-1α has been identified as key regulator of the inflammatory transcription factor NF-κB [20]. Other studies confirmed several parallels between the transcriptional regulation of hypoxia and inflammation/infection [21]-[27]. For example, a recent study suggests that NF-κB is a critical transcriptional activator of HIF-1α and that basal NF-κB activity is required for HIF-1 protein accumulation under hypoxia [28]. Similarly, studies of human pathogens have revealed that exposition of host cells to bacteria (e.g. *Bartonella henselae*) results in HIF-1α activation and VEGF secretion *in vivo* and *in vitro* [29]. Similar findings were reported when macrophages were infected with group B streptococci [17], [18]. Therefore, it is obvious that HIF-1 plays a central role in infections with human bacterial pathogens. This could have important medical implications in terms of the treatment of sepsis, as it has been shown that serum VEGF levels (known to be regulated via HIF-1) are dramatically increased in patients suffering from septicemia [30] or meningitis [31].

In addition, previous studies have found activation of HIF-1α during viral infections [32]. For example, previous studies have suggested a functional role of the hepatitis C virus in HIF stabilization [33]. Other studies found HIF-1α stabilization during hepatitis B infections [34] or during infections with the Eppstein-Barr virus [35]. Respiratory syncytial virus (RSV) is the major cause of serious lower respiratory disease in infancy and early childhood [36]. Bronchiolitis, the more severe clinical manifestation of RSV infection, is characterized by necrosis and sloughing of the respiratory epithelium and plugging of the small bronchioles with fibrin and mucus. As such, RSV is characterized by a particularly prominent inflammation of the pulmonary mucosa-both in natural and experimental infections [36]. In fact, RSV is among the most potent biological stimuli that induce the expression of inflammatory genes, including those encoding chemokines, and studies on mechanism(s) that control virus-mediated airway inflammation are currently areas of intense investigation [37]–[41]. In fact, a previous studies suggested that RSV-elicited release of nitric oxide could be associated with HIF-1α stabilization during RSV infection [42]. Therefore, we combined in vitro and in vivo approaches to study HIF-1α activation and gene-transcription during RSV infection.

## Methods

### Culture of epithelial cells

A549 cells (American Type Culture Collection, Wesel, Germany) were cultured as described previously [38]. In short, A549 were grown in F-12 HAM medium (Invitrogen, Karlsruhe, Germany) supplemented with 10% fetal bovine serum (Invitrogen, Karlsruhe, Germany) and 1% Antibiotic-Antimycotic-Solution (Sigma-Aldrich, Steinheim, Germany). L-Glutamin was adjusted to a total quantity of 2 mM. The cells were maintained under standard-conditions of 37°C in 20% O2 and 5% CO2.

### Infection with RSV

Human RSV was purified by polyethylene glycol precipitation, followed by centrifugation on 35 to 65% discontinuous sucrose gradients as described previously [43], [44]. The virus was stored in aliquots at −80°C until use. Virus titers were determined by a methylcellulose plaque assay [45]. For experiments with inactivated RSV, the virus was exposed to an UV light source for 20 minutes as described previously [43]. A549 cells were infected when they reached 70–80% confluence, using different multiplicities of infection (MOI). The virus was added immediately after removal of the culture medium in a small amount of serum-free medium for 1 h. Additional media was added and the infection was continued for indicated time periods [38].

### Protein Extraction

Supernatants were discarded and 200 µl Lysis-Buffer (1 mM Tris-HCl, 250 mM NaCl, 1 mM EDTA, Triton X 100 1%, NP40 1× Igepal Electrophoresis Reagent, Aprotinin 1 µg/ml, Leupeptin 1 µg/ml, Pepstatin 1 µg/ml, PMSF 1 mM and OV 1 mM) was added. After scraping and collecting into tubes, the cell-lysate was incubated at 4°C for 20 minutes on a rotator. Cell debris was removed by centrifugation at 13000 g for 15 minutes and discharged. Protein concentrations were determined using the BCA^TM^ Protein Assay Kit (Pierce, Bonn, Germany) according to the manufacturer's instructions.

### Nuclear Protein Extraction

Nuclear Proteins were isolated from A549 cells using a modification of methods previously described [46]. In short, cells were lysed in 500 µl cold buffer A (10 mM Hepes-KOH, pH 7.9, 1.5 mM MgCl2, 10 mM KCl, 0.5 mM Dithiothreitol (DTT), 0.2 mM phenylmethylsulfonyl fluoride (PMSF)), scraped and collected into tubes and incubated for 15 min on ice. After adding 7,5 µl solution containing 10% NP40, vortexing for 3 seconds and incubation for three minutes on ice, the tubes were centrifuged for 2 minutes at 6000 g at 4°C. The cytoplasmic proteins in the supernatant were collected and flash-frozen. Next, the pellet was resuspended in 100 µl of cold buffer B (20 mM Hepes-KOH, pH 7.9, 25% glycerol, 420 mM NaCl, 1.5 mM MgCl2, 0.2 mM EDTA, 0.5 mM DTT, 0.2 mM PMSF). After centrifugation (12000 g at 4°C for 30 minutes), the supernatant was discarded and the pellet was resuspended in 50 µl of buffer C (25% glycerol, 20 mM Hepes-KOH, pH 7.9, 420 mM NaCl, 1.5 mM MgCl2, 0.2 mM EDTA, 0.5 mM DTT, 0.2 mM PMSF, 2 mM benzamidine, 5 mg/ml leupeptin) and incubated for 45 minutes at 4°C. Cellular debris was removed by 5 minutes of centrifugation (6000 g at 4°C) and the supernatant was flash-frozen at −80°C. Protein concentrations were determined using the BCA^TM^ protein assay kit (Pierce, Bonn, Germany) as instructed by the manufacturer.

### Western blotting

Proteins were diluted in radio-immuno precipitation assay (RIPA) buffer to equivalent protein concentrations. After adding 4× Laemmli sample buffer they were immediately heated for 10 minutes at 70°C, separated on a 12% polyacrylamide gel and transferred to a nitrocellulose membrane (Polyvinylidene Difluoride, Bio-Rad Laboratories, Inc., München, Germany). Rainbow (Amersham, Buckinghamshire, UK) and MagicMark (Invitrogen, Karlsruhe, Germany) were used for size analysis and blotting control. The membranes were blocked overnight at 4°C in TBS containing 0,05% Tween and supplemented with 3% BSA and 3% skimmed milk. The membranes were then incubated in 1∶500 COX2 goat polyclonal IgG (Santa Cruz, Heidelberg, Germany) or 1∶500 anti RSV mouse IgG (Acris, Hiddenhausen, Germany) or 1∶500 FN goat polyclonal IgG (Santa Cruz, Heidelberg, Germany) or 1∶1000 beta-Actin antibody (Cell Signaling, Danvers, MA) in blocking buffer. After three washes, membranes were incubated with horse-radish-peroxidase-labelled secondary antibodies (goat anti-rabbit or donkey anti-goat or goat anti-mouse 1∶1000; Santa Cruz, Heidelberg, Germany) for 45 minutes at room temperature. The wash was repeated and proteins were detected by enhanced chemiluminescence, using the Chemiluminescent Substrate Kit (Pierce, Bonn, Germany). Western immunoblotting for HIF1-alpha was performed using 1∶500 Anti-HIF-1α rabbit polyclonal IgG (upstate, Lake Placid, NY) following the manufacturers protocol without the use of Tween.

### Enzyme-linked immunosorbent assay (ELISA) for determination of chemokines

Total protein samples of infected or non-infected A549 cells were tested for VEGF by use of a commercial ELISA kit (R&D Systems, Minneapolis, MN, USA) as instructed by the manufacturer. In short, samples were added to a 96 well microtiter plate, which was coated with murine monoclonal antibody to VEGF. The unbound protein was removed by washing and an enzyme linked polyclonal antibody specific to VEGF was added. After additional washing, substrate solution was added and incubated for 20 min. The color-reaction was stopped with stop solution and the amount of VEGF was determined by optical density of the samples by comparing the standards at 450 nm using an ELISA reader.

### Immunohistochemistry

A549-cells were cultured on glass slides (NalgeNuc International, Naperville, IL) and infected with RSV at a MOI of 3. After 24 hours, they were fixed and permeabilized for immunofluorescent staining using Cytofix/Cytoperm (PharMingen, BD-Bioscience, Heidelberg, Germany). After two washes with Perm/Wash-solution (PharMingen, BD-Bioscience, Heidelberg, Germany) the slides were blocked for 30 minutes with 5% skimmed milk in Perm/Wash-solution. Purified mouse anti-HIF1α Mab (BD Transduction Laboratories, BD-Bioscience, Heidelberg, Germany) and anti-RSV mouse IgG (Acris, Hiddenhausen, Germany) were diluted 1∶100 in Perm/Wash-solution and the slides were incubated for 30 minutes. Normal mouse and normal rabbit control IgG in a dilution of 1∶200 were used. After two washes with Perm/Wash-solution, the slides were incubated for 30 minutes with the secondary antibodies (Alexa Fluor 488 goat anti-rabbit IgG and Alexa Fluor 594 goat anti-mouse IgG, Invitrogen, Karlsruhe, Germany) in Perm/Wash. The slides were embedded with a reagent containing DAPI (Invitrogen, Karlsruhe, Germany) for staining of the nuclei. Fluorescence was visualized with a confocal laser scanning microscope (Leica, Bensheim, Germany).

### Reverse Transcription Polymerase Chain Reaction Analysis

Realtime RT-PCR (iCycler; Bio-Rad Laboratories Inc., Hercules, California, USA), was used to verify COX2, FN-1, VEGF and CD73 transcript levels of RSV-infected A549 cells. After infection with RSV with an infection dose of MOI3 for 24 h, total RNA was isolated using the RNA II Kit (Macherey & Nagel, Düren, Germany) and real-time RT-PCR was performed as described previously [47]–[52]. The PCR reaction contained 10 pM each of the sense primer 5′-AAA CCT CAG CTC AGG ACT GC-3′ and the antisense primer 5′-GGC ACT AGC CTC TTT GCA TC-3′ for COX2, sense primer 5′-AAG GAA GGG GAA GAA CAG GA-3′ and the antisense primer 5′-GGC AGA GCT GAT GGA ATC TC-3′ for CD73, sense primer 5′-TTG CCT TGC TGC TCT ACC TC-3′ and the antisense primer 5′-AGC TGC GCT GAT AGA CAT CC-3′ for VEGF, sense primer 5′-AGG CTC AGC AAA TGG TTC AG-3′ and the antisense primer 5′-TCG GCT TCC TCC ATA ACA A-3′ for FN1. The primer set for COX2, FN1 and VEGF was amplified using increasing numbers of cycles of 95°C for 15 sec, 58°C for 30 sec, 72°C for 10 sec, and a final extension of 72°C for 1 minute. The primer set for CD73 was amplified using increasing numbers of cycles of 95°C for 15 sec, 60°C for 30 sec, 72°C for 10sec, and a final extension of 72°C for 1 minute. Human beta-actin (sense primer, 5′-GGT GGC TTT TAG GAT GGC AAG-3′; and antisense primer, 5′-ACT GGA ACG GTG AAG GTG ACA G-3′) was used as control.

### Stable repression of HIF-1α by siRNA

Repression of HIF-1α by siRNA was achieved based on a modification of methods previously described [26], [53]–[56]. In short, a hairpin primer with the sequence 5′-ACCTCGCTGACCAGTTATGATTGT-GATCAAGAGTCACAATCATAACTGGTCAGCTT-3′ and 5′-CAAAAAGCTGACCAG-TTATGATTGTGACTCTTGATCACAATCATAACTGGTCAGCG-3′ corresponding to position 2666-2685 of the HIF1α gene was selected. A549-cells were transfected using electroporation, followed by selection with G418 (1 mg/ml). The control cell line was transfected with a non-specific control psiRNA-hH1 neoscr plasmid.

### Blood Gas Analysis

Blood gas analysis was performed to assess oxygen partial pressure in supernatants of uninfected or infected A549 cells. The cells were cultured and infected at a MOI of 1 or 5. One hour after infection the cell-culture flasks were filled up with serum free media and were sealed gas-tight. Analysis of the supernatants was performed immediately after removal via the I-STAT Analyzer (Abbott, Wiesbaden, Germany) at different time points as described previously [19], [52].

### Infection of mice with RSV and extraction of lung nuclear proteins

Female, 6- to 8-week-old BALB/c mice were purchased from Harlan (Houston, Texas, USA) and were housed in pathogen-free conditions in the animal research facility of the University Texas Medical Branch (UTMB), Galveston, Texas, in accordance with the National Institutes of Health and UTMB institutional guidelines for animal care. The Institutional Animal Care and Use Committee approved this protocol. Cages, bedding, food, and water were sterilized before use. Under light anesthesia, female, 6-8 weeks old BALB/c mice were infected intranasally with RSV at 1×10^7^ plaque-forming units (PFUs), diluted in sterile PBS for a total inoculation volume of 50 µl. As mock treatment, control mice were inoculated in the same way with an equivalent volume of sucrose diluted in PBS. At the indicated time points after infection (12, 24 and 48 h) mice were anesthetized with an intraperitoneal injection of ketamine and xylazine before the thoracic cavity was opened [39]. Lungs were then removed, quick frozen in liquid nitrogen and stored at −80°C until nuclear protein was isolated. Nuclear proteins were isolated from the lung tissue using a modified method described by Bohrer and colleagues [46]. Lung tissue was homogenized in 5 ml ice-cold Buffer A (10 mM 2-hydroxyethyl-piperazine N′-2-ethanesulfonic acid [Hepes]–KOH, pH 7.9, 1.5 mM MgCl_2_, 10 mM KCl, 0.5 mM dithiothreitol [DTT], 0.2 mM phemylmethyl sulfonyl fluoride [PMSF], 0.6% nonident P40 [NP-40]) and centrifuged at 350×g, 4°C for 30 seconds. The supernatant was kept on ice for 5 minutes and centrifuged for 5 minutes at 6,000×g at 4°C, and the pellet was resuspended in 200 µl Buffer B (10 mM Hepes–KOH, pH 7.9, 1.5 mM MgCl_2_, 10 mM KCl, 1.2 M sucrose, 0.5 mM DTT, 0.2 mM PMSF). After centrifugation (13,000×g, 4°C, 30 minutes), the pellet was resuspended in 100 µl Buffer C (20 mM Hepes–KOH, pH 7.9, 1.5 mM MgCl_2_, 420 mM NaCl, 0.2 mM ethylenediamine-tetraacetic acid, 0.5 mM DTT, 0.2 mM PMSF, 2 mM benzamidine, 5 µg/ml leupeptin, 25% glycerol), incubated on ice for 20 minutes, and centrifuged (6,000×g, 4°C, 2 minutes). The supernatant was quick frozen in aliquots at −80°C. HIF-1α stabilization was determined by Western blot analysis as described previously [56].

### Statistical Analysis

Data collection and statistical analysis was performed using Microsoft Excel (Microsoft Office Professional Edition 2003) and Graph Pad Prism (GraphPad Software Inc., Prism 4 for Windows Version 4.03). All presented values were calculated as the mean from at least three separate experiments. The results in the control and the viral infection group were analyzed and compared by utilizing the Unpaired Student t Test and the Mann-Whitney Nonparametric Test. For all statistical analyses a P value<0.05 was considered significant.

## Results

### Immunolocalization of HIF-1α during RSV infection in vitro

**Figure 1:**
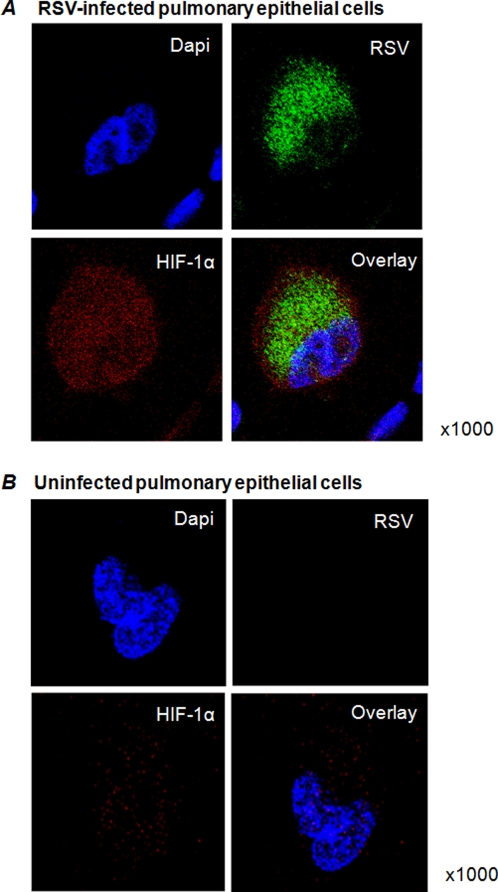
HIF-1α during RSV infected of pulmonary epithelia. (A) ∼1,5×10^5^ A549-cells were seeded on glass slides and infected with RSV (multiplicity of infection, MOI 3). After 24 h they were fixed, permeabilized and incubated with anti-HIF1α and anti-RSV IgG as primary antibodies. Alexa Fluor 488 and Alexa Fluor 594 were used for staining. In addition, slides were counter-stained with Dapi. The cells were visualized with confocal laser scanning microscopy. Uninfected cells were used as controls (B).

Recent evidence revealed that numerous parallels exist between inflammation and hypoxia, including changes in barrier function or inflammatory cell recruitment [5], [6], [55], [57]–[60]. In addition, recent studies have revealed that during infections with human pathogens, HIF-1α is activated [19], [29]. Previous studies demonstrated that RSV infections are characterized by a particularly prominent inflammation of the pulmonary mucosa-both in natural and experimental infections-and RSV is among the most potent biological stimuli that induce the expression of pro-inflammatory genes [36]. Therefore, we hypothesized that HIF-1α is stabilized during infections with RSV and may contribute to RSV-associated changes in gene expression. For our studies, we used A549 cells, a cell line derived from an alveolar cell carcinoma of the lung. As first step, we performed immunohistochemical staining with antibodies for RSV (green) or HIF-1α (red) using confocal laser scanning microscopy (Figure 1A). As counterstaining for the nuclei we used dapi staining (blue). As shown in Figure 1A, RSV infected cells also stained positive for HIF-1α, with localization of HIF-1α both in the cytosole and the nuclei. This is consistent with other studies demonstrating that HIF-1α is present in the cytosole and in the nucleus [61]. In contrast, uninfected A549 cells only had a very week signal for HIF-1α (Figure 1B). Isotype controls and staining of infected A549 cells with secondary antibody alone were negative (data not shown). Taken together, these data reveal that during RSV infection, HIF-1α accumulates in the cytosole and the nucleus of infected pulmonary epithelia, suggesting HIF-1α activation during RSV infection in vitro.

### HIF-1α protein is stabilized during RSV infection

**Figure 2:**
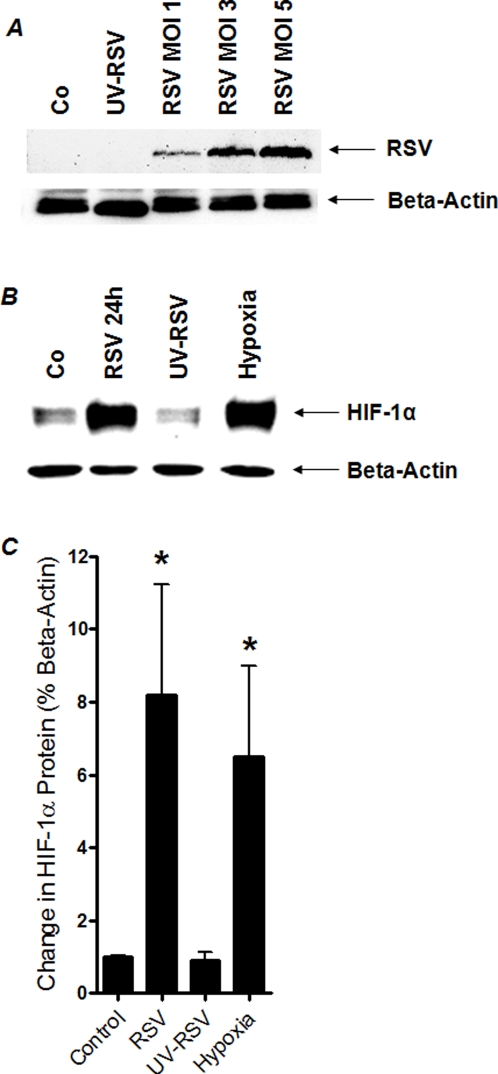
HIF-1α protein measurements during RSV infection in vitro. (A, B) Cultured pulmonary epithelia (A549) were grown to 80% confluency, infected with intact (multiplicity of infection, MOI 1, 3 or 5) or UV-inactivated RSV (MOI 3). In other studies A549 cells were exposed over 24 h to ambient hypoxia (2% oxygen). Cells were lysed and nuclear proteins were isolated, and Western immunoblotting for RSV G-protein (A) or HIF1α was performed. Uninfected cells were used as control (Co). The same blots were probed for β-actin expression as a control for protein loading. A representative blot of 3 is shown, in addition to densitometric analysis of HIF-1α protein levels relative to β-actin (C;*P<.01, different from control, n  =  3).

After having shown by confocal laser scanning microscopy that HIF-1α is stabilized during RSV infection, we next used Western blot analysis to confirm these results with a more quantitative approach. Here, we first confirmed successful infection of A549 cells using different infection doses (MOI1-5). As shown in Figure 2A, we found a close correlation of virus load with RSV G-protein after 24 h of infection (Figure 2A). In contrast, RSV pre-exposed to UV light source as previously described for RSV inactivation [43] showed no signal for intracellular RSV G-protein. Uninfected cells were used as negative control. As next step, we measured HIF-1α during RSV infection by Western blot analysis. These studies revealed an 8.2±5.2-fold increase in HIF-1α protein compared to non-infected A549 cells (Figure 2B and C, relative to beta-actin, MOI of 3). Interestingly, this HIF-response was completely attenuated when using UV-treated RSV virus (0.9±0.42-fold HIF-1α protein relative to beta-actin, Figure 2 B and C). In additional control studies, we exposed A549 cells to ambient hypoxia (2% oxygen over 24 h), which was associated with a robust increase in HIF-1α protein (6.5±4.3-fold increase in HIF-1α). Taken together, these studies reveal robust stabilization of HIF-1α during infection with life RSV in vitro.

### HIF-dependent genes are induced following RSV infection

**Figure 3:**
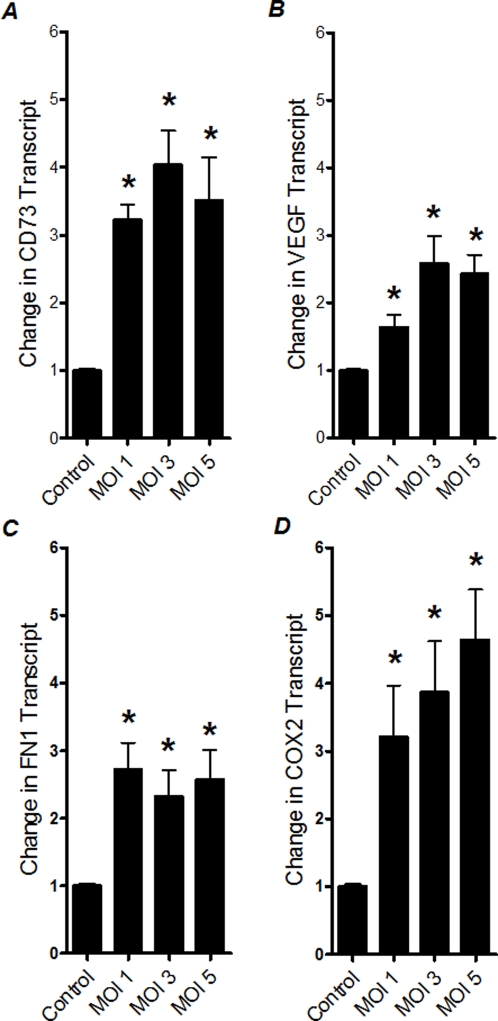
Transcript levels of HIF-1-dependent genes following RSV infection. Total RNA was isolated from RSV-infected (multiplicity of infection, MOI 1, 3 and 5) or non-infected A549 cells (control) and (A) CD73, (B) VEGG, (C) FN1, (D) COX2 mRNA levels were determined by real-time RT-PCR. Data were calculated relative to internal housekeeping gene (β-actin) and are expressed as fold increase over uninfected control-cells ±SEM at each infection dose (*P<0.05, different from uninfected control-cells).

**Figure 4:**
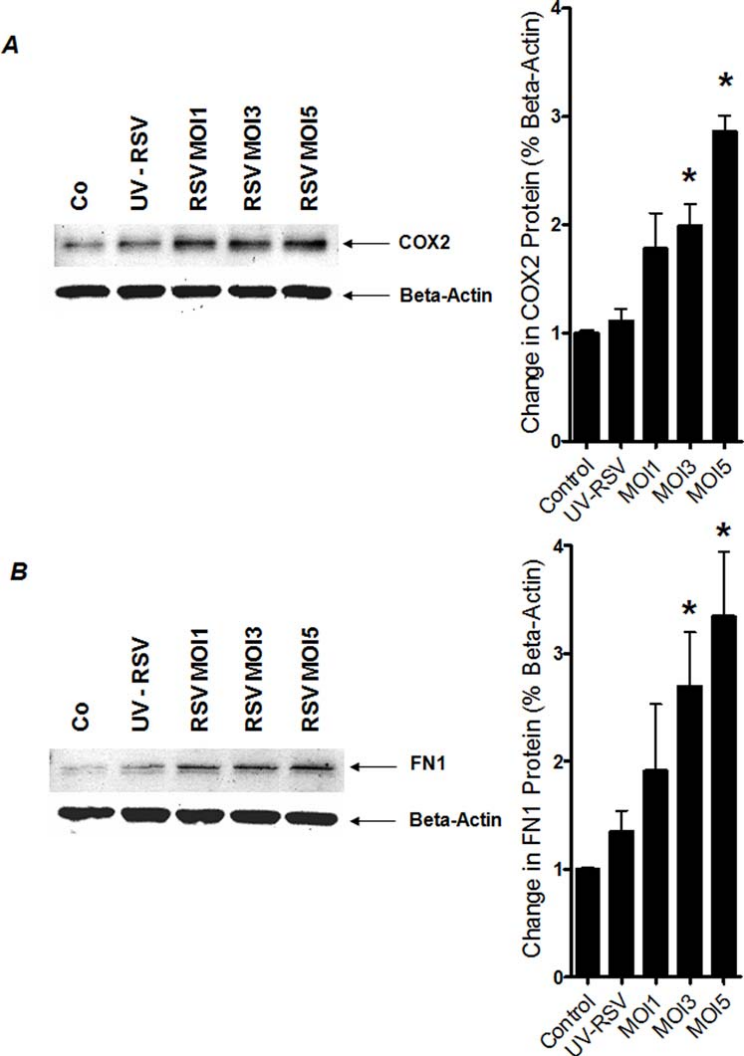
Protein levels of HIF-1-dependent genes following RSV infection. Total protein was isolated from RSV-infected (multiplicity of infection, MOI 1, 3 and 5) or non-infected A549 cells. Protein levels were determined by Western blot. The same blots were probed for β-actin expression as a control for protein loading. In addition, densitometric analysis of protein levels relative to β-actin were performed. Data are expressed as fold increase over uninfected control-cells ±SEM at each infection dose. (A) COX2; (B) FN1 (*P<0.01, different from control, n  =  3).

After having demonstrated HIF-1α protein stabilization during infection with RSV, we next pursued functional consequences of HIF-1α in transcriptional gene induction during RSV infection. For this purpose, we performed expressional studies of known HIF-1-dependent genes during RSV infection. Thus, we measured transcript levels of CD73 [62], VEGF [63], Fibronectin1 (FN1) [64] and COX2 [65] after 24 h of RSV infection of A549 pulmonary epithelial cells using different RSV infection doses (MOI1-5). As shown in Figure 3, analysis of transcript levels by real-time RT-PCR revealed induction of all tested HIF-1 target genes. Extensions of these findings at the protein level by Western blot confirmed significant induction of COX2 or FN1 protein levels (Figure 4A and B). Similarly, measurements of VEGF in the supernatants from RSV infected pulmonary epithelial cells revealed significantly elevated levels of VEGF (data not shown). Taken together, these studies demonstrate induction of HIF-1α -depedendent genes during RSV infection in vitro.

### HIF-dependent gene expression during RSV infection in pulmonary epithelial cells following siRNA repression of HIF-1α

**Figure 5:**
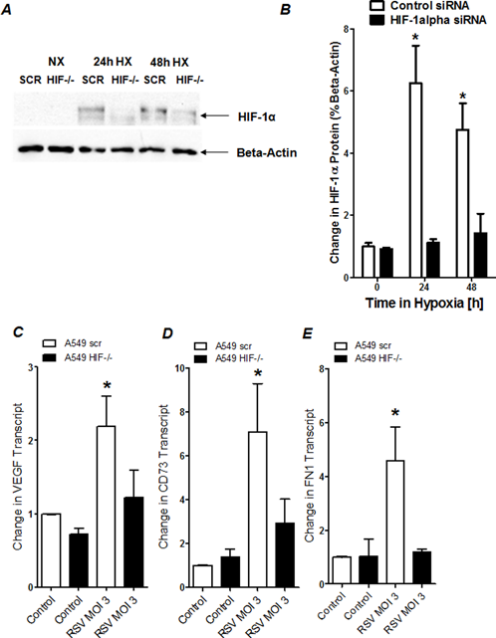
Transcript levels of HIF-dependent genes following HIF-1α siRNA repression during RSV infection. (A) HIF-1α protein levels in A549 cells following hairpin siRNA repression of HIF-1α (HIF-/-; A549 cells transfected with control siRNA:SCR). Cells were grown to 80% confluency and exposed to normoxia or hypoxia (2% oxygen) over indicated time period. Nuclear proteins were isolated and Western Blot analysis was performed for HIF-1α. The same blots were probed for β-actin expression as a control for protein loading. A representative blot of 3 is shown, in addition to densitometric analysis of HIF-1α protein levels relative to β-actin (B;*P<0.01, different from control, n  =  3).(C, D, E) Total RNA was isolated from RSV-infected (multiplicity of infection, MOI 3) or non-infected A549 following HIF-1α repression (A549 HIF-/-) or transfection with control siRNA (A549 scr). (C) VEGF, (D) CD73, (E) FN1 transcript levels were determined by RT-PCR. Data were calculated relative to internal housekeeping gene (β-actin) and are expressed as fold increase over uninfected control-cells ±SEM (*P<0.05).

To demonstrate a functional role of HIF-1α in the observed induction of HIF-dependent genes induction during RSV infection, we next pursued HIF-1α-loss-of-function studies. For this purpose, we generated a pulmonary epithelial cell line (A549 cells) with stable repression of HIF-1α. This was achieved via hairpin siRNA technique as we have done previously in other cell lines [26], [53]–[56]. To demonstrate effective repression of HIF-1α in these cell lines, we utilized a model of ambient hypoxia. For this purpose, we exposed these cells to hypoxia over 24 or 48 h (2% oxygen), while growth-synchronized control cells were maintained at room air (21% oxygen). These studies revealed significant accumulation of HIF-1α protein in control transfected cells in conjunction with attenuated HIF-1 α stabilization in HIF-1 α-siRNA-transfected A549 cells (Figure 5A and B). We utilized this model to directly test the functional role of HIF-1α in transcriptional modulation during RSV infection. Here, we measured transcript levels of control transfected or HIF-1α-targeted pulmonary epithelia. These studies revealed significant induction of HIF-1-target genes (VEGF, CD73 or FN1) in control cells, while these responses were abolished in HIF-1α-targeted pulmonary epithelia. Taken together, these studies suggest a functional role of HIF-1 α in transcriptional induction of HIF-1α -targeted genes during infection with RSV.

### Influence of UV-inactivation of RSV on HIF-dependent gene induction

**Figure 6:**
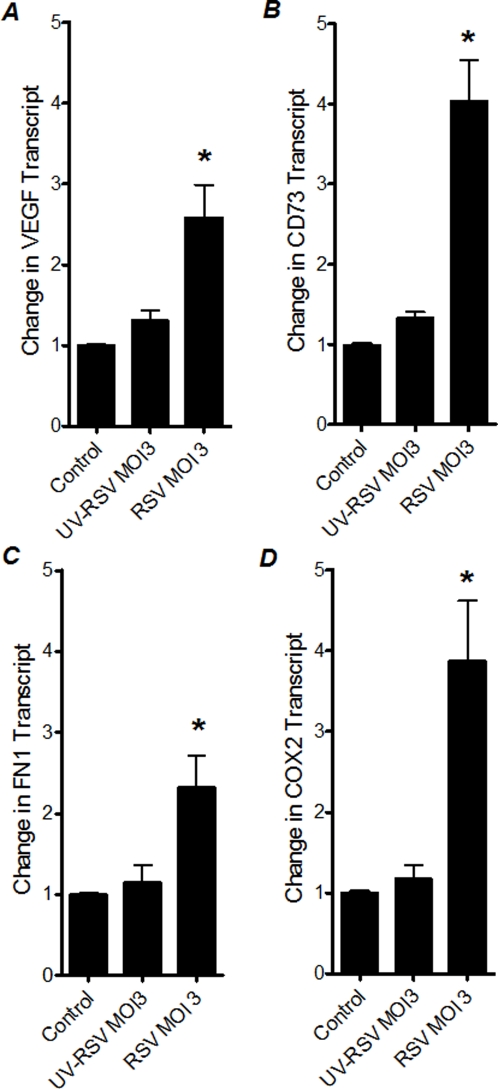
Transcript levels of HIF-dependent genes following infection with inactivated RSV. Total RNA was isolated from uninfected, RSV-infected or UV-inactivated RSV infected A549 cells. (A) VEGF, (B) CD73, (C) FN1, (D) COX2 transcript levels were determined by real-time RT-PCR. Data were calculated relative to internal housekeeping gene (β-actin) and are expressed as fold increase over uninfected control-cells ±SEM at each infection dose (*P<0.05, different from uninfected control-cells).

In view of the above results, we hypothesized that only intact RSV is capable of HIF-1 α stabilization and induction of HIF-1α target genes. Therefore, we next investigated the effects of UV-inactivated virus on the HIF-1 α target genes. For this purpose, we measured VEGF, CD73, FN1 and COX2 transcript levels in A549 cells that were infected with intact or with UV-inactivated RSV (Figure 6 A-D). Consistent with our studies above, we found significant induction of HIF-1α target genes in RSV infected A549 cells. In contrast, induction of HIF-1α target genes was completely abolished after similar infection doses with UV-inactivated RSV. Taken together, these studies suggest that only functional RSV virus, capable of intracellular replication–is necessary to cause HIF-1α-dependent gene induction.

### HIF1α stabilization after RSV infection occurs independent of hypoxia

**Figure 7:**
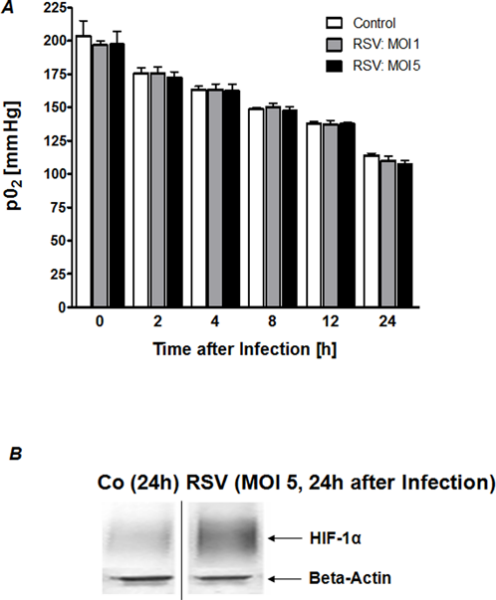
(a) *Measurements of oxygen partial pressures (pO_2_) in the supernatants of RSV infected pulmonary epithelia.* (A) A549 cells were cultured and infected at a multiplicity of infection (MOI) of 1 or 5 in gas-tight sealed flasks. Oxygen partial pressure was measured in the supernatants at indicated time points following infection. (B) A samples was assessed for HIF-1α protein levels by Western blot 24 h after RSV infection or control conditions. Blots were probed for β-actin expression as a control for protein loading

Previous studies of infection and inflammation have revealed significant changes in metabolic supply and demand. For example, studies of murine colitis revealed convincing evidence that the mucosal surface is prone to inflammation-associated hypoxia [58], [66]. Our above studies showed that intracellular uptake and binding of RSV appears to be necessary for HIF-1α dependent gene induction. Therefore, we hypothesized that changes in metabolic supply and demand ratios during RSV infection may accompany RSF-infection and RSV-associated tissue hypoxia could lead to HIF-1α activation. To address this hypothesis in an experimental setting, we measured oxygen partial pressures (pO_2_) values in the supernatants from RSV infected pulmonary epithelia or controls that were maintained in an oxygen impermeable culture system. As shown in Figure 7A, no differences in PO_2_ values were observed between experimental groups. In fact, neither supernatant from control nor RSV-infected pulmonary epithelia showed significant degrees of hypoxia. Consistent with these findings, and as shown in Figure 7B, Western blot analysis confirmed that HIF-1α was not stabilized in control cells maintained under the above cell culture conditions at 24 h. Similarly, HIF-1α stabilization was not observed at earlier time points (date not shown). In contrast, we observed significant HIF-1α stabilization in RSV-infected pulmonary epithelia. Taken together, these studies suggest oxygen independent stabilization of HIF-1α during RSV infection.

### HIF-1 α is stabilized during murine RSV infection *in vivo*

**Figure 8:**
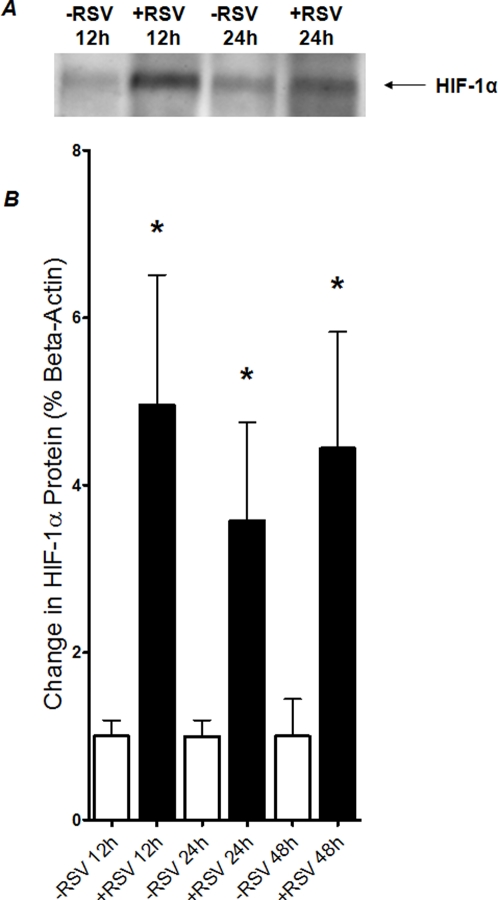
HIF-1α protein during murine RSV pneumonia in vivo. Female, 6- to 8-week-old BALB/c mice were inoculated intranasally with purified RSV at 1×10^7^ plaque-forming units (PFUs), diluted in sterile 0.9 % sodium chloride for a total inoculation volume of 50 µl. As mock treatment, control mice were inoculated in the same way with an equivalent volume of sucrose diluted in 0.9 % sodium chloride. Lungs were removed at indicated time-points, and HIF-1α protein levels were determined by Western blot analysis (A) or quantified by densitometry, relative to beta-actin (B; *p<0.01).

As proof of principle for these concepts in vivo, we compared the influence of RSV infection on pulmonary HIF-1α stabilization using a previously described model [38]–[41]. This mouse model shows close similarity to the pathogenesis of RSV-induced lower airway disease in humans. In fact, recently established that the experimental infection of BALB/c mice with highly purified preparations of RSV A, at a dose of 10^7^ PFU, induces a severe inflammatory response in lung tissue as early as 24 h after intranasal inoculation [39]. Lung inflammation was characterized by an excess of monocytes/macrophages, lymphocytes, and to a lesser extent, neutrophils surrounding bronchioles and vessels, with evidence of the involvement of alveolar spaces [39]. In the present studies, female BALB/c mice were inoculated intranasally with purified RSV. In control experiments, BALB/c mice matched in age, gender and weight were inoculated in the same way with an equivalent volume of vehicle. At the indicated time points after infection, mice were anesthetized, lungs were shockfrozen and HIF-1α was determined by Western blot analysis. As shown in Figure 8A and B, HIF-1 α was stabilized with RSV infection at all measurement time points. Taken together, these data confirm our *in vitro* findings and suggest that during murine RSV pneumonia HIF-1α is stabilized.

## Discussion

Many studies during the last decade have demonstrated a central role of HIF-1 in mammalian oxygen homeostasis [3]. However, more recently many studies have also demonstrated a role of HIF-1α in the transcriptional coordination during inflammation and infection [6], [32], [54], [67]. In fact, previous studies have revealed that HIF-1α can be stabilized during infections with human pathogens via oxygen-dependent [67] or oxygen independent [54] pathways. In the present studies we pursued HIF-1α stabilization and gene-transcription during infection with RSV–one of the most potent biological stimuli to induce an inflammatory milieu [38]–[41]. In initial studies of cultured pulmonary epithelia infected with RSV revealed stabilization of HIF-1α protein. Moreover, transcription of known HIF-1α target genes was induced following RSV infection, while siRNA-dependent repression of HIF-1α abolished these responses. However, infection with RSV was not associated with increased oxygen consumption or cellular hypoxia, suggesting that HIF-1α stabilization and HIF-dependent gene induction during RSV infection occurs in an oxygen-independent fashion. Finally, studies of murine RSV pneumonia revealed significant HIF-1α stabilization throughout the course of the disease and suggest that RSV-associated HIF-1α activation also occurs in vivo.

Previous studies of HIF-1α during inflammation and infection have found oxygen-independent activation of HIF-1α during infections with human pathogens. For example, a recent study on molecular mechanisms of how bacteria activate HIF-1α found a role of bacterial siderophores in HIF-1α activation during infection with Enterobacteriaceae [54]. Here, the authors studied HIF-1α activation and HIF-1α -dependent gene induction in Peyer's patches that were analyzed after orogastric infection with Yersinia enterocolitica and orogastric Y enterocolitica infection in mice with a conditional deletion of HIF-1α [66] in the intestine. These studies demonstrated that infection of mice with Y enterocolitica led to functional activation of HIF-1α in Peyer's patches. Moreover, mice with conditional deletion of HIF-1α in the intestinal epithelium showed a significantly higher susceptibility to orogastric Y enterocolitica infections, suggesting HIF-1α activation as a host defense mechanism in this model. Additional studies with Y enterocolitica, S enterica subsp enterica, or E aerogenes, and, moreover, application of their siderophores (yersiniabactin, salmochelin, aerobactin) caused a robust, dose-dependent HIF-1α response in human epithelia and endothelia, independent of cellular hypoxia. Taken together, such studies demonstrate a role for bacterial siderophores in hypoxia-independent activation of HIF-1α during infection with human pathogenic bacteria [54].Similarly, previous studies on viral infections with RSV have demonstrated induction of HIF-1α in primary human bronchial epithelial cells via a nitric-oxide-dependent pathway [42]. Other studies have identified a crosstalk between viral genes and the HIF-1α pathway during infections with the human herpesvirus 8 (HHV-8) [32], [68]. Kaposi's sarcoma-associated herpesvirus (KSHV or HHV-8) is the etiological agent of Kaposi's sarcoma, a highly vascularized, endothelial-derived tumor. A direct role for KSHV-mediated induction of angiogenesis has been proposed based upon the nature of the neoplasia and various KSHV gene overexpression and infection model systems. These studies revealed that KSHV infection of endothelial cells induces mRNA of HIF-1α and HIF-2α. While HIF is classically activated posttranscriptionally, these studies demonstrate that both alpha-subunits are up-regulated at the transcript level by KSHV infection. Here, the transcriptional activation of HIF leads to a functional increase in HIF activity under normoxic conditions, as shown utilizing luciferase reporter assays and HIF-dependent gene expression.

From the present studies it remains unclear whether HIF-1α activation during RSV infection represents a host-defense mechanism or is an essential part of the disease pathogenesis enabling virus uptake or replication. While some studies have identified a host-protective role of HIF-1α during inflammation [25]–[27], [53], [54], [69] or infections [54], other studies have found a contribution of HIF-1α activation in growth and survival of human pathogens. For example, Toxoplasma gondii is an obligate intracellular protozoan pathogen. Recent studies revealed that genes mediating cellular responses to hypoxia were upregulated in Toxoplasma -infected cells but not in cells infected with another intracellular pathogen, Trypanosoma cruzi [70], [71]. The inducible expression of these genes is controlled by the HIF-1α. Additional studies revealed that Toxoplasma infection rapidly increased the abundance of the HIF1α and activated HIF-1α reporter gene expression and survival was severely reduced in cells targeted for HIF-1α [72]. These studies also suggested that while HIF-1α was not required for parasite invasion, HIF-1α was required for parasite cell division and organelle maintenance, indicating that Toxoplasma activates HIF-1α and requires HIF-1α for growth and survival at physiologically relevant oxygen levels [70]–[72].

Taken together, the present studies reveal oxygen-independent stabilization of HIF-1α during RSV infection *in vitro and in vivo*. Future challenges will include the determination whether such responses elicited during RSV infections are host-protective or host-detrimental. In addition, it will be critical to gather a more thorough understanding of the mechanisms of HIF-1α induction during RSV infection (e.g. the role of RSV elicited TLR signaling in HIF stabilization) or the contribution of RSV-associated nitric oxide release [42]. Ongoing studies are currently testing HIF-activation or HIF-inhibition in different settings of medical therapy and novel therapeutics will soon become available in patient care to inhibit or to activate the HIF-1α pathway. Such compounds may comprise a novel approach during RSV infections.
